# Surface Coating of Nickel-Titanium (Ni-Ti) Pediatric Rotary File Using Graphene Oxide: A Scanning Electron Microscopy Analysis

**DOI:** 10.7759/cureus.66632

**Published:** 2024-08-11

**Authors:** Kuheli Panja, Victor Samuel A, Vivek N, Kavitha Ramar

**Affiliations:** 1 Pediatric Dentistry, Sri Ramaswamy Memorial (SRM) Kattankulathur Dental College and Hospital, SRM Institute of Science and Technology, Kattankulathur, IND; 2 Oral and Maxillofacial Surgery, Sri Ramaswamy Memorial (SRM) Kattankulathur Dental College and Hospital, SRM Institute of Science and Technology, Kattankulathur, IND; 3 Pedodontics and Preventive Dentistry, Sri Ramaswamy Memorial (SRM) Kattankulathur Dental College and Hospital, SRM Institute of Science and Technology, Kattankulathur, IND

**Keywords:** kedo-sg pediatric file, sem analysis, electrophoresis deposition method, surface chemistry, graphene oxide, ni-ti endodontic instrument

## Abstract

Background: The effectiveness of endodontic files relies significantly on the characteristics of the outermost layer, which can be greatly improved through suitable surface treatments and appropriate coatings. Graphene-based materials (GBMs) have been utilized to fabricate nanocomposite coatings aimed at improving surface characteristics and mechanical behavior, including resilience, sustainability, oxidation resistance, solidity, and traction.

Aim: This research aims to study the surface topography of a nickel-titanium (Ni-Ti) pediatric rotary file coated with graphene oxide (GO) using a scanning electron microscope (SEM).

Methods: The study utilized Ni-Ti pediatric rotary instruments that were 16 mm long and had the same ISO tip size of #25. The Ni-Ti pediatric rotary files had a titanium oxide coating that needed to be removed for the application of the GO coating. The GO coating was applied to the files using an electrophoretic deposition (EPD) procedure. Data were gathered to evaluate the surface topography and structural profiles of the GO-coated endodontic files through SEM analysis.

Results: SEM imaging showed that the GO coatings consisted of numerous layers of GO sheets, which were uniformly and thoroughly applied to the endodontic instrument. A substantial portion of the GO layers aligned with neighboring layers along the edges, creating a continuous structure.

Conclusion: GO coatings were effectively applied to Ni-Ti endodontic instruments using EPD. The deposition of the GO coating is consistent throughout the surface of the Ni-Ti rotary instrument.

## Introduction

The field of dental materials has seen remarkable advancements in recent years, with notable improvements in the tools and configurations employed. The efficacy of dental materials is largely dependent on their surface properties, which can be further enhanced through the application of specialized treatments and coatings. Surface engineering in this context often involves the incorporation of cutting-edge technologies, such as nanotechnology, to optimize the materials' characteristics [1]. One prominent advancement in dental materials is the widespread use of nickel-titanium (Ni-Ti) alloys in biomedical applications. These alloys are prized for their exceptional properties, making them suitable for various medical devices, including vascular stents, staples, catheters, orthodontic wires, and endodontic instruments [2]. While the fundamental principles underlying endodontic instruments have not undergone significant changes, the past decades have witnessed a proliferation of specialized designs, resulting in increasingly complex armamentariums. This diversification of endodontic instrument configurations has been driven by the ongoing pursuit of enhanced performance and clinical outcomes [1,2].

Surface modifications and coatings are paramount in enhancing surface quality, mitigating corrosion, and minimizing bodily reactions. Despite concerted efforts to apply various polymer coatings to Ni-Ti alloys, achieving successful adhesion has proven to be a significant challenge [1,2]. Graphene, a material consisting of a film of sp2 carbon atoms arranged in a two-dimensional hexagonal lattice, has emerged as a potential solution to this problem [3]. Researchers [3-8] have been employing graphene-based materials (GBMs) to develop nanocomposite coatings to improve surface characteristics and mechanical properties. These enhancements include increased resilience, sustainability, oxidation resistance, solidity, and traction. This development constitutes a noteworthy advancement in the fields of surface engineering and materials science. The combination of graphene with a range of components and synthetic materials allows for the creation of nanoparticle-reinforced polymers suitable for ecological, antimicrobial, biosensing, and surface film purposes [4,6,9,10]. Scientists [7,8,10] have explored various methods for creating graphene oxide (GO) coatings as surface modifications. The widely employed method for producing uniform coatings of graphene composites is electrophoretic deposition (EPD). These coatings exhibit outstanding mechanical and surface properties, making them highly suitable for diverse applications.

The primary purpose of implementing these surface modifications is to enhance the functionality of endodontic files in dentistry. The use of Ni-Ti rotary files for the mechanical preparation of primary teeth was first introduced by Barr et al. in 2000 [11]. The study found several advantages to using Ni-Ti rotary files for root canal preparation in primary teeth. These advantages include cost-effectiveness, speed, and consistently achieving uniform and predictable fillings. The use of Ni-Ti instruments in curved canals allows for the preservation of the original anatomy of primary root canals, primarily due to the design and flexibility of the files. This characteristic enables them to closely conform to the irregular and tortuous canals of the primary teeth [12]. Despite these advantageous properties, one significant drawback of the rotary file is its high incidence of fracture within the canal [2]. To address and mitigate this limitation, GO coatings are being deposited on the Ni-Ti rotary files and studied in this work to enhance the structural and functional properties of the rotary files.

Scientific research plays a crucial role in recognizing and understanding the surface properties of coatings applied to various materials. To the best of the authors' knowledge, there is currently no scientific evidence available regarding the quality of coatings on endodontic instruments. This study aims to address this gap by examining the surface topography of GO nanocomposite-coated Ni-Ti pediatric file systems utilizing scanning electron microscopy (SEM).

## Materials and methods

Tested Ni-Ti pediatric rotary instruments

The Ni-Ti pediatric rotary file system (Kedo-SG rotary files, Reeganz Dental Care Pvt. Ltd., Chennai, India) of 16 mm length and identical ISO tip size #25 (D1) were selected for this study.

Removal of titanium oxide coating from endodontic files

**Figure 1:**
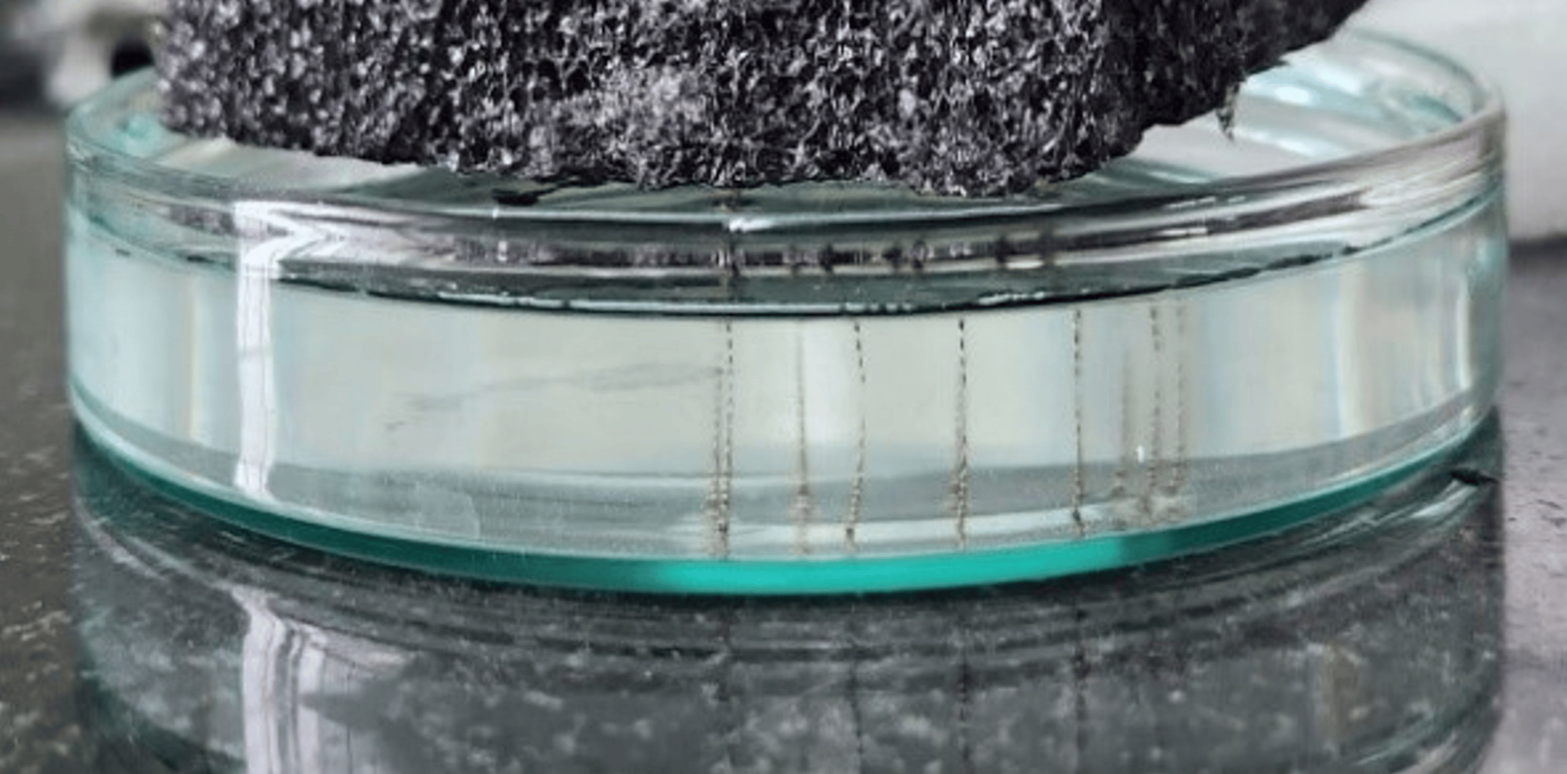
Setup for titanium oxide coating removal from the endodontic files after stabilization on an ethylene vinyl acetate foam sheet and dipped in medical-grade 0.5 mL of nitric acid and 0.5 mL of sulfuric acid in 10 mL water

**Figure 2:**
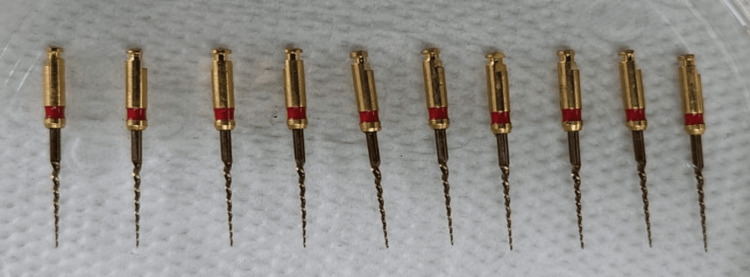
Endodontic files after the titanium oxide coating removal procedure

The pediatric rotary files (N=10) under examination were characterized by a titanium oxide coating. To facilitate the removal of this coating, a solution was prepared comprising 0.5 mL of nitric acid, 0.5 mL of sulfuric acid, and 10 mL of water, with the total volume adjusted to 100 mL. The prepared solution was subsequently transferred to a borosilicate glass Petri dish. The Ni-Ti endodontic files were positioned on an ethylene vinyl acetate foam sheet and immersed in the aforementioned solution for a duration of 15 seconds to effectuate the removal of the titanium oxide coating (Figure 1). Following the etching process, the endodontic files were extracted from the solution, subjected to thorough rinsing with distilled water, and subsequently dried at a temperature of 800 °C for a period of 15 minutes utilizing a hot air oven (Figure 2).

EPD method for GO coating on rotary files

**Figure 3:**
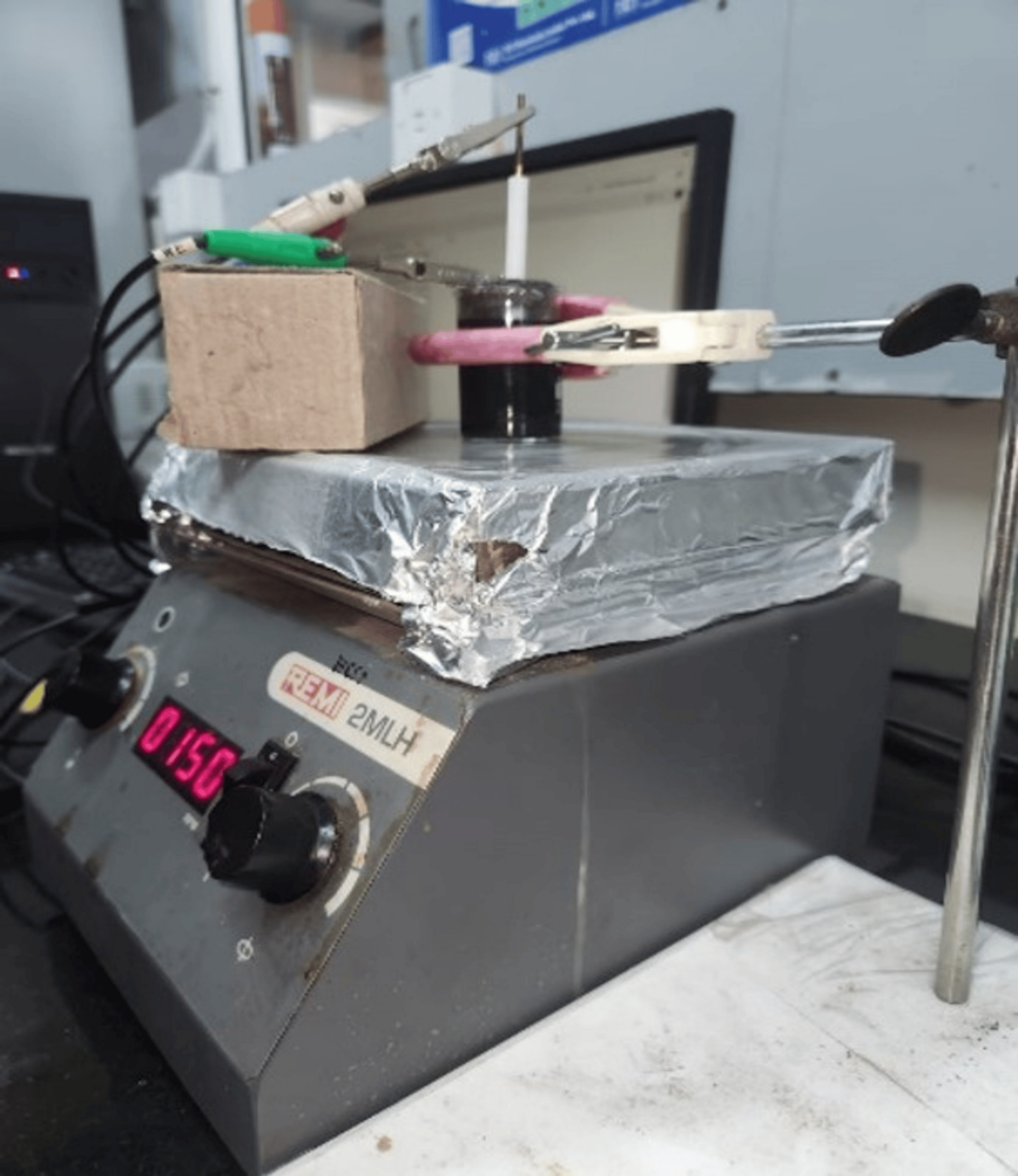
Electrophoretic deposition process for the deposition of graphene oxide on endodontic files

EPD was used to coat the etched endodontic files with GO. A 25 mL borosilicate glass beaker was filled with 24 mL of a reduced graphene oxide (rGO) solution, prepared by dispersing 1 g of rGO powder in 100 mL of deionized water. The mixture was then subjected to ultrasonication for three hours. The endodontic files (N=10) were individually subjected to the coating process. An electromagnetic stirrer was positioned at the bottom of the beaker to ensure uniform distribution of the rGO solution. The temperature of the apparatus was maintained at 40 °C throughout the procedure. Platinum foil served as the cathode, while the etched file functioned as the anode. These electrodes were positioned 15 mm apart. The deposition of GO was carried out for a duration of 10 minutes at a constant voltage of 10 V, regulated by an electronic potentiometer (Figure 3). Following the deposition process, the GO-coated endodontic files were thoroughly rinsed with distilled water. The coated files were subsequently dried at 80 °C for 15 minutes.

SEM analysis

SEM has established itself as one of the most efficient microscopy techniques, providing simplicity in operation and delivering high spatial resolution. The incorporation of analytical techniques can furnish valuable supplementary insights into the characteristics of endodontic instruments after GO deposition.

**Figure 4:**
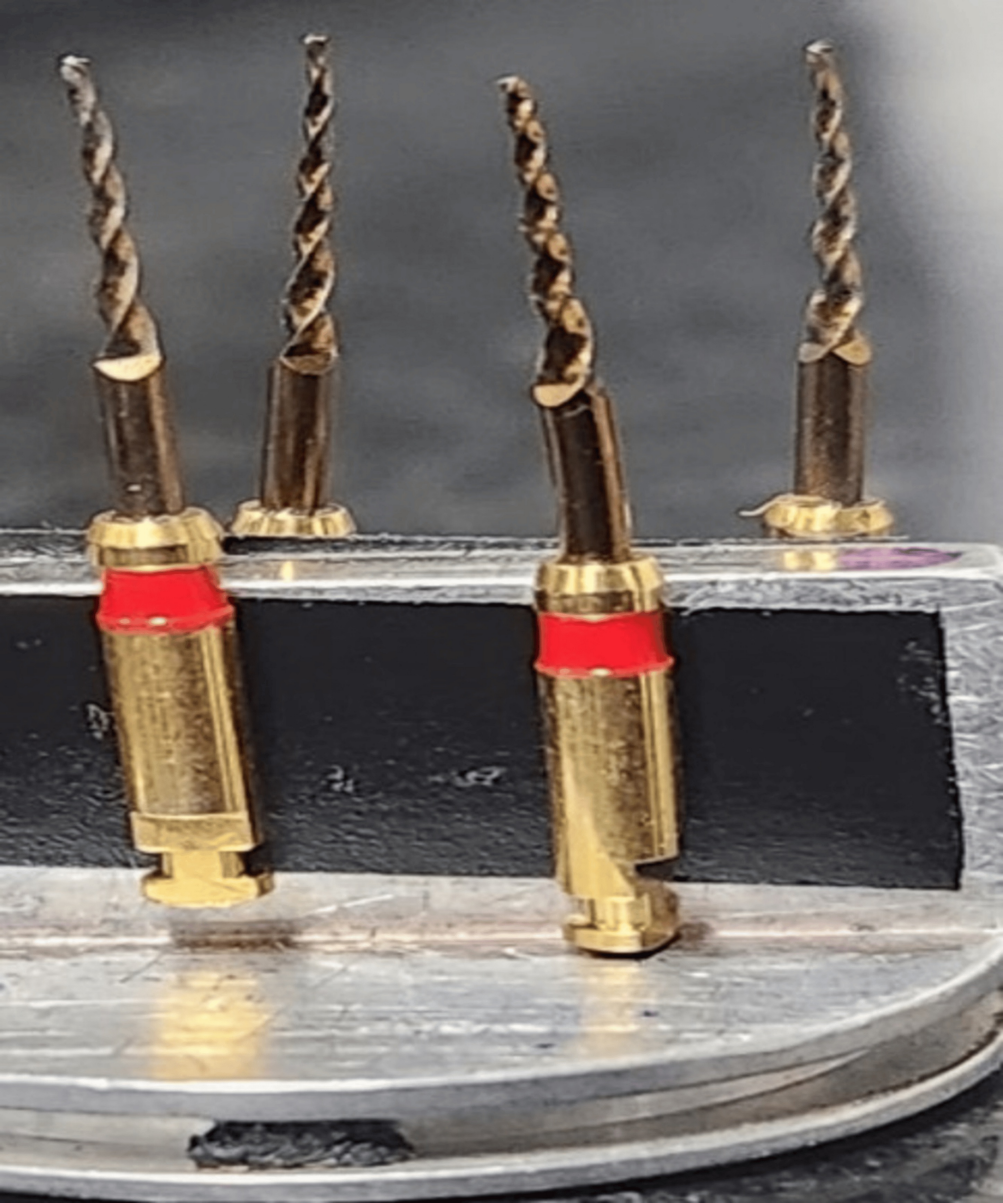
Endodontic files mounted on metallic stubs for SEM analysis SEM: scanning electron microscopy

The instruments were meticulously prepared for SEM analysis by mounting them on metallic stubs. The specimens were positioned with a precise vertical separation of 10 mm between each instrument (Figure 4). The examination protocol focused on three key areas of each instrument: the tip, the cutting surface, and the shaft. These regions were observed at a magnification of 250×. The arrangement of the specimens on the stubs followed a systematic order, with the etched endodontic files placed in the first row and the GO-coated endodontic files situated in the second row.

To conduct a comprehensive analysis of the surface topography of the GO-coated endodontic files, additional examinations were performed at higher magnifications. These included observations at 6500×, 12500×, and 100000×, allowing for a detailed study of the surface characteristics imparted by the GO coating. This multi-scale approach to SEM analysis enabled a thorough evaluation of the morphological changes induced by the GO coating process on the endodontic files.

The SEM micrographs were systematically recorded in a structured worksheet. The primary focus of the evaluation was surface topography, specifically examining the structural profiles of the GO-coated endodontic files. The subsequent analysis and discussion of the results primarily centered on the material parameters related to the chemical composition, as determined through the comprehensive SEM analysis. This methodical approach allowed for a thorough and objective assessment of the GO coating's characteristics and its integration with the endodontic file surface.

## Results

**Figure 5:**
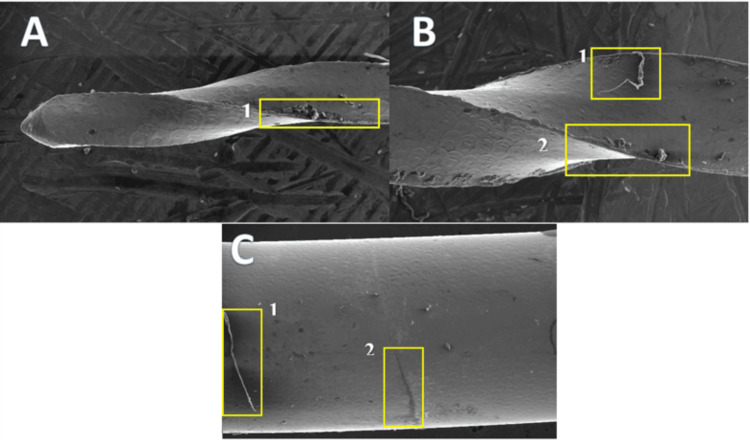
Titanium oxide coating removal of the endodontic file at 250× magnification A: The tip of the file, where (1) shows roughness on the cutting edge of the file. B: The cutting edge of the file, where (1) shows inorganic debris on the file and (2) shows an irregular surface on the cutting edge of the file. C: The shaft of the file, where (1) shows inorganic debris on the file and (2) shows a cracked surface on the file

The surface morphology of the endodontic files was meticulously examined utilizing high-resolution scanning electron microscopy (HR-SEM, Thermo Scientific Apreo S, Waltham, Massachusetts) operating at 30 kV. This study scrutinized both pre-coated and GO-coated endodontic files across various sections of the instruments. The SEM analysis disclosed that the pre-coated files exhibited numerous voids and a lack of uniformity throughout their length. Specifically, at the tip of the pre-coated file, only a few isolated, irregular particles were detected (Figure 5A). The cutting edge of these files displayed irregular voids and inorganic debris (Figure 5B). Furthermore, inorganic debris and a cracked surface were observed along the shaft of the instrument (Figure 5C).

**Figure 6:**
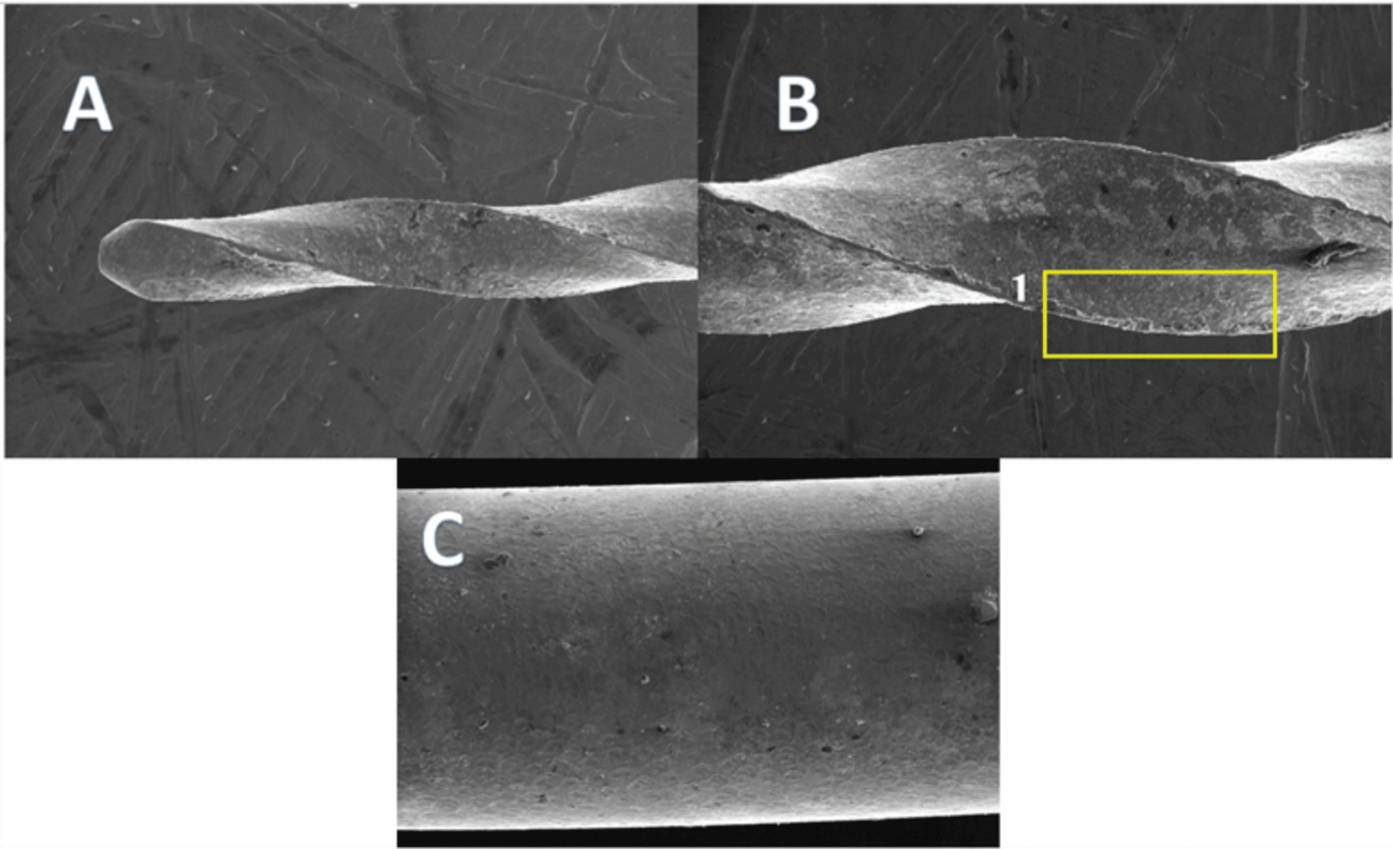
GO-coated endodontic file at 250× magnification A: The tip of the file. B: The cutting edge of the file, where (1) shows a more regular surface on the cutting edge of the file. C: The shaft of the file GO: graphene oxide

**Figure 7:**
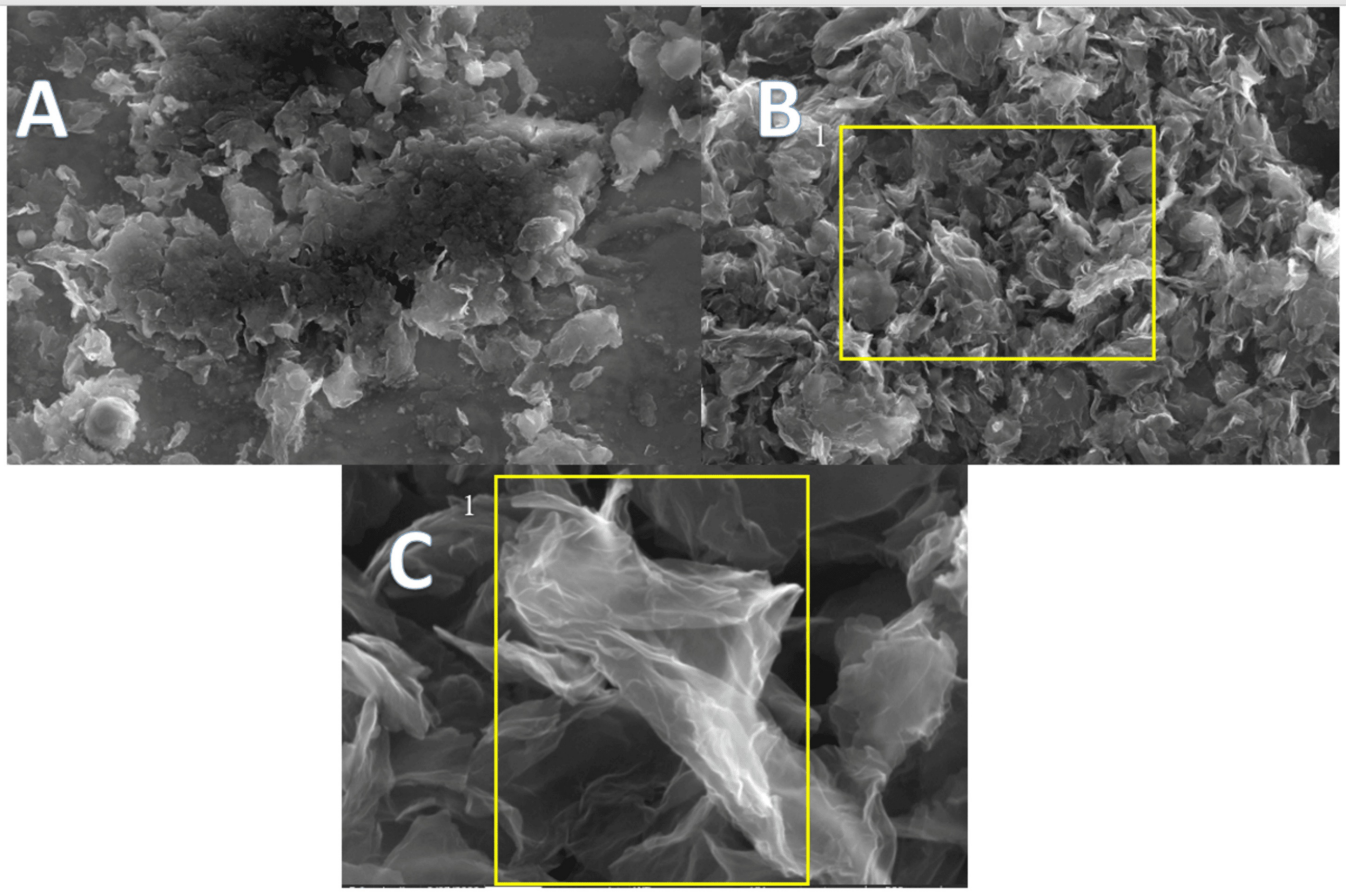
GO-coated endodontic file A: 6500× magnification. B: 12,000× magnification, where (1) shows porous nest-like GO deposits. C: 100,000× magnification, where (1) shows GO flakes GO: graphene oxide

The HR-SEM analysis at 250× magnification revealed that the endodontic files exhibited a more regular and homogeneous surface following GO coating (Figures 6A, 6C). Post-coating, the cutting edge of the endodontic file displayed a smoother surface compared to the pre-coated counterpart (Figure 6B). At higher magnifications, the GO-coated endodontic instruments demonstrated stratified GO films characterized by high homogeneity, comprehensive coverage, and multiple stacked layers (Figure 7A). These multiple layers arise due to the strong van der Waals forces between the graphene layers, which cause them to be attracted to each other. A significant number of these GO layers aligned with adjacent layers at the edges, creating an uninterrupted structure (Figure 7A). The coated file was entirely enveloped with inorganic aggregates of GO (Figure 7B). Under 12,000× magnification, HR-SEM images revealed a porous, nest-like structure within the multiple GO sheets on the GO-coated endodontic files (Figure 7B). At 100,000x magnification, mineral aggregates appeared as dense flakes on the coated file (Figure 7C).

## Discussion

The design matrix and materials employed in endodontic files are crucial, as they greatly impact the characteristics of the file system. Clinicians must be aware of these properties and the distinctions in materials utilized in emerging technologies [13]. The current in vitro work has studied the surface modifications of the pediatric rotary file after GO coating. Several key factors influence the properties and chemical composition of the alloy [14-16].

The advancement of endodontic treatment, particularly with the integration of Ni-Ti instruments, has been substantial. However, the risk of fracture persists due to various factors. These factors include intrinsic characteristics of the file, such as cross-sectional dimension, size, taper, and alloy composition, all of which can influence different mechanical properties. Furthermore, the anatomy of the canal also plays a significant role in affecting these properties [17].

The frequent use of instruments during endodontic procedures often results in surface degradation post-instrumentation. This degradation underscores the critical need for instruments capable of withstanding multiple uses, despite manufacturers generally advocating for single-use. The endodontic files employed in this study are composed of Ni-Ti alloy, characterized by a triangular profile and a blunt tip. Additionally, these files possess a negative rake angle and a variably variable taper. Each file measures 16 mm in length, with 12 mm of cutting blades constituting the working area [18]. Previous studies have demonstrated that these files enhance obturation quality in pediatric root canals, thereby justifying their selection for this study.

GO films can be synthesized using various methods, including chemical vapor deposition, EPD, and spin coating. To enhance the surface characteristics of endodontic instruments, GO coating via the EPD process was employed in this study. The EPD method was selected based on the findings of several researchers, including Zhang et al. (2018), Singh et al. (2013), and Vesna et al. (2014), who concluded that this technique is simple, benign, and capable of reproducibility [19]. Recent evidence has demonstrated that GO-effective films applied to the surface of endodontic instruments exhibit several advantageous properties, such as (i) reduced friction, (ii) anticorrosive effects, and (iii) anti-cariogenic bacterial activity [20-22]. The EPD process employed in this study has demonstrated its effectiveness in producing a uniform GO coating on the endodontic Ni-Ti files, and the reproducibility of the EPD procedure was successfully attained in the laboratory environment.

In 2020, Srimaneepong et al. [23] demonstrated that Ni-Ti alloy laminated with GO displayed a smooth and homogeneous morphology and exhibited superior corrosion resistance compared to bare Ni-Ti alloy. These findings corroborate our results indicating a uniform and smooth coating on the Ni-Ti endodontic files, as evaluated under SEM analysis across the complete length of the file.

The study conducted by Alshahrani et al. in 2020 [19] reported that single-layer GO, with a thickness of approximately 100 nm, exhibited nanoplatelet characteristics under SEM analysis. Furthermore, their research findings suggested that GO enhanced adhesive properties. However, our SEM analysis yielded contrasting results, indicating that the GO coatings on the files are comprised of multiple layers.

Rokaya et al. also conducted a study in 2019 [7] revealing the nanocomposite GO coating exhibited enhanced mechanical strength and decreased friction coefficients. These findings suggested that the GO-coated Ni-Ti alloy may be more suitable for biomedical applications due to its improved mechanical properties and reduced friction, potentially leading to better performance in biomedical settings. Based on SEM images, it was observed that the coatings were homogeneous and free from defects, indicating their durability and stability.

However, further investigation into the longevity and resilience of electrodeposited graphene coatings is necessary before considering their sustainable clinical applications. The limitation of this study is that it does not equip the determination of the coating thickness through the analysis provided. A potential area for future research could involve examining the cross-section of the endodontic file after it has been coated.

## Conclusions

This study established the deposition of a uniform layer of GO coating on the surface of Ni-Ti rotary instruments using EPD, as evidenced through SEM analysis. The GO coatings of endodontic files indicate the potential for enhancing fracture resistance and improving the overall mechanical properties of these files during endodontic procedures. Future research is needed to explore the characteristic properties of endodontic files coated with GO.
